# Diversity and evolutionary history of endogenous retroviruses in the genome of *Manis pentadactyla*

**DOI:** 10.1128/spectrum.03203-24

**Published:** 2025-09-30

**Authors:** Tiancheng Zhang, Juan Xu, Hongfeng Yang, Chenglin Zhou, Wen Zhang

**Affiliations:** 1 Institute of Critical Care Medicine, The Affiliated People's Hospital, Jiangsu University 196541 , Zhenjiang, China; 2 Department of Laboratory Medicine, School of Medicine, Jiangsu University, Zhenjiang, China; 3 Clinical Laboratory Center, The Affiliated Taizhou People’s Hospital of Nanjing Medical University, Taizhou, China; Shandong First Medical University, Jinan, Shandong, China

**Keywords:** endogenous retrovirus, phylogenetic analysis, evolution, *Manis pentadactyla*

## Abstract

Endogenous retroviruses (ERVs), remnants of ancient viral infections integrated into host genomes, serve as invaluable molecular fossils for studying viral evolution. In this study, we performed a genomic analysis of the Chinese pangolin (*Manis pentadactyla*), identifying novel full-length endogenous retroviruses, designated as *Manis pentadactyla* ERVs (MPERVs). MPERVs span three retroviral genera: *Alpha*-, *Beta*-, and *Gamma-retroviruses*. Using genomic screening and phylogenetic analysis, we classified MPERVs and reconstructed their evolutionary history, uncovering evidence of complex recombination events and cross-species transmission. Estimated insertion times for MPERVs range from very recent to 18.38 million years ago. MPERVs exhibit diverse structural features, notably including conserved retroviral domains and functional motifs and highlighting their preservation across extensive evolutionary periods. These findings shed light on the evolutionary dynamics of ERVs in Chinese pangolin and suggest the potential for expanded host ranges among certain retrovirus genera.

**IMPORTANCE:**

Endogenous retroviruses are unique viruses distinguished by the fact that they are retained as part of the host genome after an exogenous retrovirus infects the host. The Chinese pangolin, as a host with a long independent evolutionary history, likely holds valuable insights in its genome regarding retrovirus endogenization and transmission. In this study, we identified the footprints of exogenous retroviruses from three different genera in the pangolin genome: *Alpharetrovirus*, *Betaretrovirus*, and *Gammaretrovirus*. Additionally, by calculating the integration times of the pangolin’s endogenous retroviruses and analyzing the domains of the three main functional proteins (GAG, POL, and ENV), we found that the insertions are relatively young. This suggests that these endogenous retroviruses infected the Chinese pangolin long before their endogenization. This study represents the exploration of endogenous retroviruses in the Chinese pangolin genome, expanding our understanding of endogenous retroviruses in mammals. Furthermore, our findings provide new evidence for the phenomenon of the cross-species transmission of retroviruses prior to endogenization.

## INTRODUCTION

Retroviruses are a highly diverse and widespread group of RNA viruses. A defining feature of their replication cycle is the integration of the viral genome into the host chromosomes, where they may persist as genomic components. The remnants of such ancient retroviral infections are termed endogenous retroviruses (ERVs) (1). ERV fragments identified in vertebrate genomes serve as invaluable molecular fossil records for reconstructing the long-term evolutionary dynamics of retroviruses. Comparative analysis of ERVs across species enables the reconstruction of their distribution patterns and cross-species transmission history. Recent advances in genomic sequencing technologies have facilitated ERV detection across diverse taxa. Notably, discoveries, such as koala-derived ERVs in Australian bats (2), provide direct evidence of cross-species transmissions, while ERVs identified in diverse marine and terrestrial mammals like killer whales (3), polar bears (4), and kangaroos (5) broaden our understanding of retroviral host range and evolutionary timelines. Collectively, these findings offer novel perspectives on the evolutionary history of ERVs.

The Chinese pangolin (*Manis pentadactyla*), a phylogenetically distinct member of the order *Pholidota* (family: *Manidae*), is a representative species of an ancient mammalian lineage that diverged from *Carnivora* approximately 71 million years ago. This extended period of independent evolution makes it an exceptional model for investigating ERV evolution and historical retroviral spread among mammals. Although previous studies have identified various viruses in pangolins, including canine *parvovirus* (6), novel *Pestivirus* strain (7), and severe acute respiratory syndrome coronavirus 2 (8), research specifically focusing on endogenous retroviruses in the Chinese pangolin genome remains limited. To date, only two ERV-related findings have been reported: the integration of a bat-derived retrovirus into the Chinese pangolin genome (9) and the discovery of an ERV sequence in the Chinese pangolin lung tissue using a viral metagenomics approach (10). Notably, comprehensive genome-wide annotation and analysis of ERVs in this species are lacking.

In this study, we discovered and characterized three novel pangolin endogenous retrovirus lineages through genomic screening, which we named *Manis pentadactyla* endogenous retroviruses (MPERVs). Phylogenetic and comparative genomic analyses revealed the complex evolutionary history of these MPERVs, elucidating retroviral-host coevolution within this unique lineage.

## RESULTS

### Identification and classification of endogenous retrovirus elements in the Chinese pangolin genome

**Fig 1:**
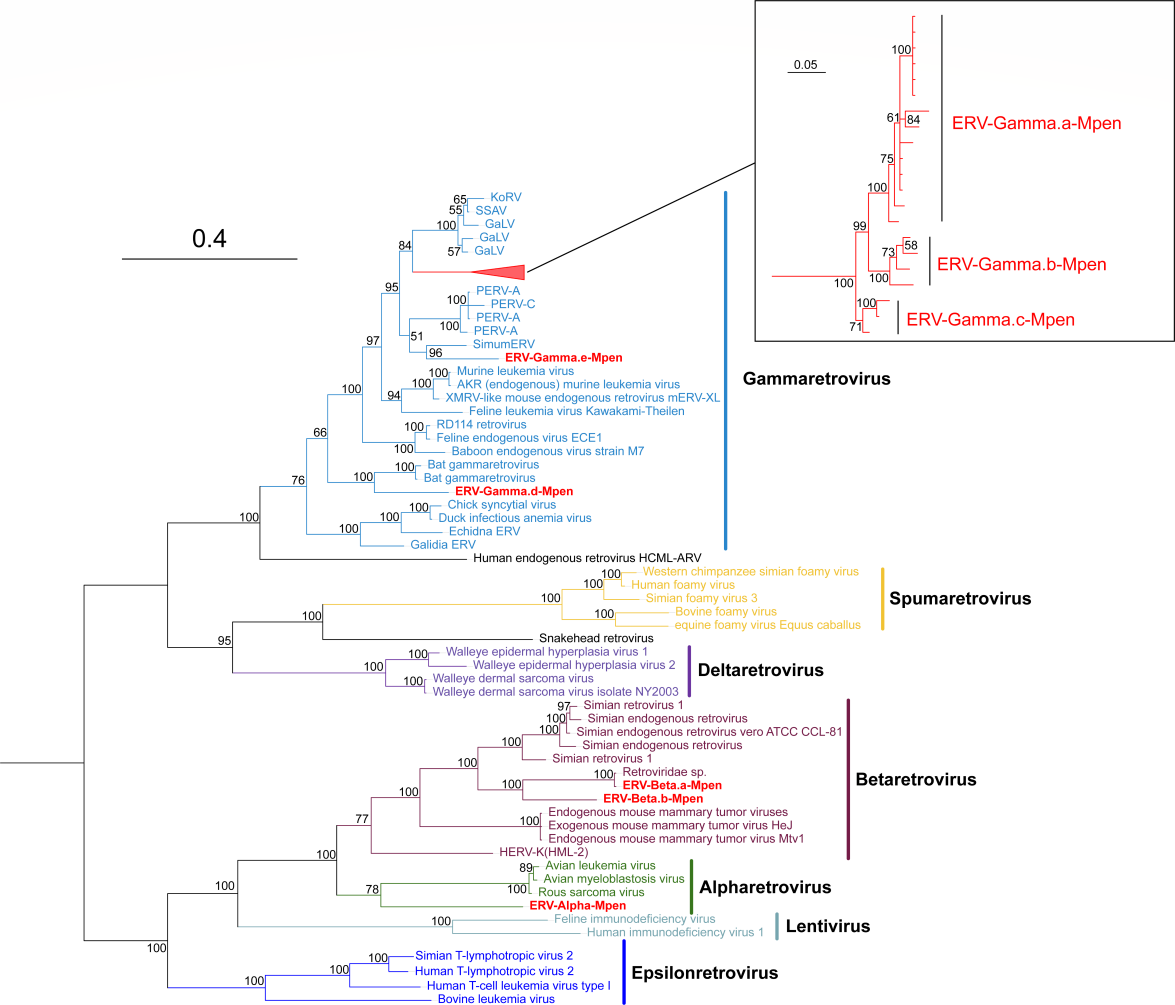
Unrooted phylogeny of retroviruses and MpenERVs. The tree was inferred from the reverse transcriptase (RT) protein alignment. The newly identified MpenERVs are labeled in red. The scale bar indicates the number of amino acid changes per site. The tree is midpoint rooted for clarity only. Lineages ERV-Gamma.a-Mpen, ERV-Gamma.b-Mpen, and ERV-Gamma.c-Mpen are displayed in the zoom-in panel. Viruses not labeled with colors represent unclassified retroviruses.

Using the POL protein sequences of known retroviruses as probes, we performed BLASTx searches against the Chinese pangolin genome (assembly GCF_030020395.1) and preliminarily identified 27 candidate elements. To confirm whether these elements belong to ERVs, a phylogenetic tree was constructed based on representative reverse transcriptase (RT) sequences from retroviruses (Fig. 1). These elements clustered within well-supported clades (posterior probability [PP] > 0.75) of *Alpha-*, *Beta-*, and *Gammaretroviruses*, confirming their classification as MPERVs. To ensure comprehensive detection of ERV elements, we conducted additional screening using the tBLASTn algorithm with the same POL protein queries, thereby mitigating potential omissions due to sequence degradation or nonsense mutations. Redundant hits were manually removed, and the remaining sequences were integrated with the initial candidates. To characterize the novel MPERVs, we used LTR_Finder/LTR_Harvest to identify paired long terminal repeat (LTR) sequences flanking the elements, as well as retrovirus-specific primer binding sites (PBS) and polypurine tracts (PPT). Full-length elements containing paired LTRs and internal regions were then used as queries in BLASTn searches to identify lineage-specific copies. Following the nomenclature guidelines for ERVs (11) and based on the topology of the phylogenetic tree, we classified the identified elements into distinct lineages: ERV-Gamma.an-Mpen (*n* = 1 to 83), ERV-Gamma.bn-Mpen (*n* = 1 to 30), ERV-Gamma.cn-Mpen (*n* = 1 to 7), ERV-Gamma.dn-Mpen (*n* = 1 to 62), ERV-Gamma.en-Mpen (*n* = 1 to 27), ERV-Alpha.n-Mpen (*n* = 1 to 13), ERV-Beta.an-Mpen (*n* = 1 to 21), and ERV-Beta.bn-Mpen (*n* = 1 to 4).

### Genomic features of MPERVs

**Fig 2:**
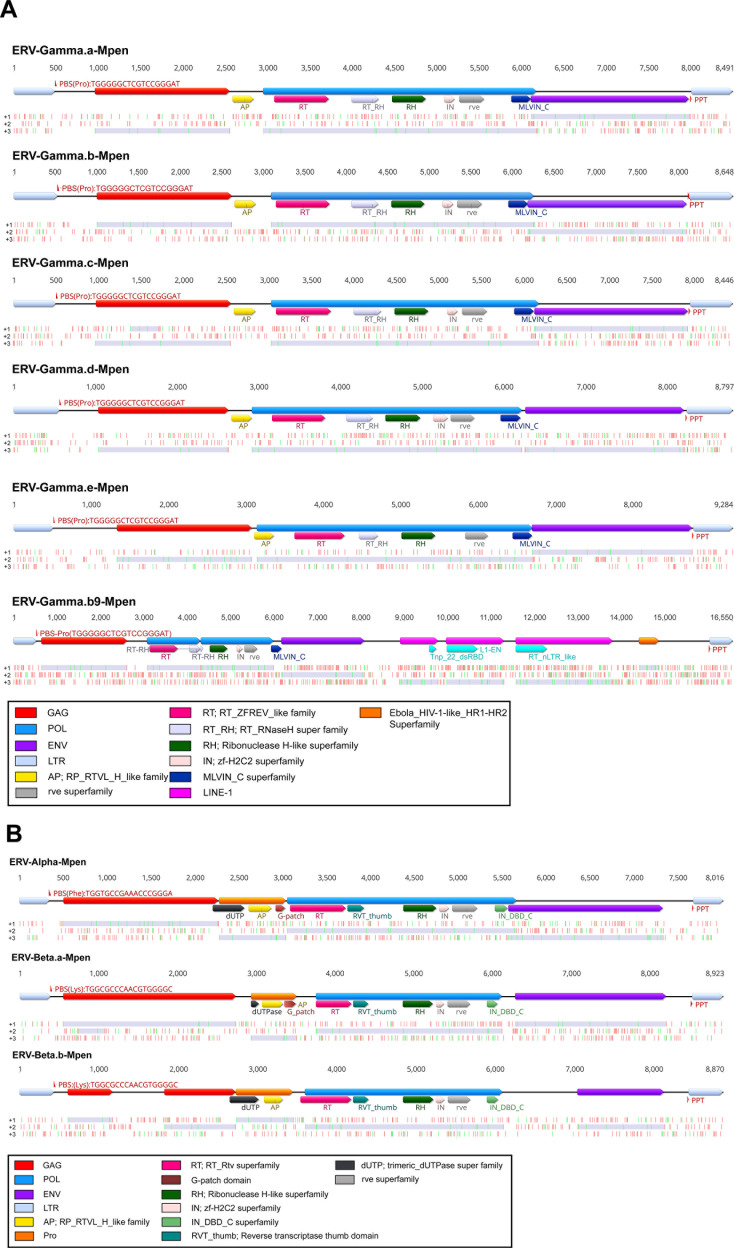
Genomic organization of MPERVs. The genome of MPERVs is depicted using lines and boxes drawn to scale. (**A**) ERV-Gamma-Mpen family. (**B**) ERV-Alpha-Mpen and ERV-Beta-Mpen families. The distributions of stop (red) and start (green) codons in three forward frames (from top to bottom, +1, +2, and +3) are shown under a genomic schematic diagram for each consensus genome. Open reading frames (ORFs) longer than 180 nucleotides are filled in. LTR, long terminal repeat; GAG, group-specific antigen gene; POL, polymerase gene; ENV, envelope gene; Pro, protease; RT, reverse transcriptase; RH, Rnase H; INT, integrase; AP, HIV retropepsin-like domain; LINE-1, long interspersed nuclear elements-1.

We generated consensus sequences for each MPERV lineage to characterize their genomic architecture (Fig. 2). All ERV-Gamma.(a-e)-Mpen and ERV-Alpha-Mpen elements contained primer binding sites (PBS-Pro and PBS-Phe) identical to previously reported sequences (12). Similarly, ERV-Beta.a-Mpen and ERV-Beta.b-Mpen exhibited PBS-Lys sequence matching that of the Mason-Pfizer monkey virus (MPMV; PBS-Lys-1,2) (13). Structural analysis revealed that ERV-Gamma.(a-e)-Mpen, ERV-Alpha-Mpen, and ERV-Beta.a-Mpen retained canonical retroviral organization, including paired long terminal repeats (LTRs) and the three major retroviral genes (GAG, POL, and ENV) (Fig. 2). Overall, the POL protein length varies among different lineages. Due to lineage-specific differences among retroviruses, the newly identified ERVs classified under the Gamma family exhibit longer POL proteins (ranging from 1,048 to 1,185 amino acid [aa] residues in length), closely resembling those of MLV (murine leukemia virus, 1,091 aa in length) and RD114 virus (1,015 aa in length). In contrast, ERV-Beta.b-Mpen encoded a truncated ENV protein likely resulting from mutational degradation.

Through the conserved domain database (CDD) search, we identified characteristic retroviral domains in MPERVs (Fig. 2). The ERV-Gamma-Mpen lineage exhibited typical retroviral domains including: aspartic protease (AP) domain (cl06095), reverse transcriptase (RT) domain (cl40470), RT_RNaseH superfamily (pfam ID 17917), RNase_H_like superfamily (cl14782), zf-H2C2 (IN) superfamily (cl07828), integrase core (rve) domain (pfam ID 00665), and MLVIN_C domain (pfam ID 18697). Additionally, the trimeric dUTPase superfamily (cl00493), HIV_retropepsin_like (AP) superfamily (cl11403), G_patch superfamily (cl02611), reverse transcriptase thumb (RVT) domain (pfam ID 06817), and IN_DBD_C superfamily (cl02895) were identified in ERV-Alpha-Mpen and ERV-Beta-Mpen. It is noteworthy that ERV-Gamma.b9-Mpen exhibits unique structural characteristics with an almost complete LINE-1 element insertion existing between its ENV and 3′-LTR regions (with less than 80% nucleotide similarity to other ERV-Gamma.b-Mpen sequences) and likely disrupts the previously encoded ENV.

**Fig 3:**
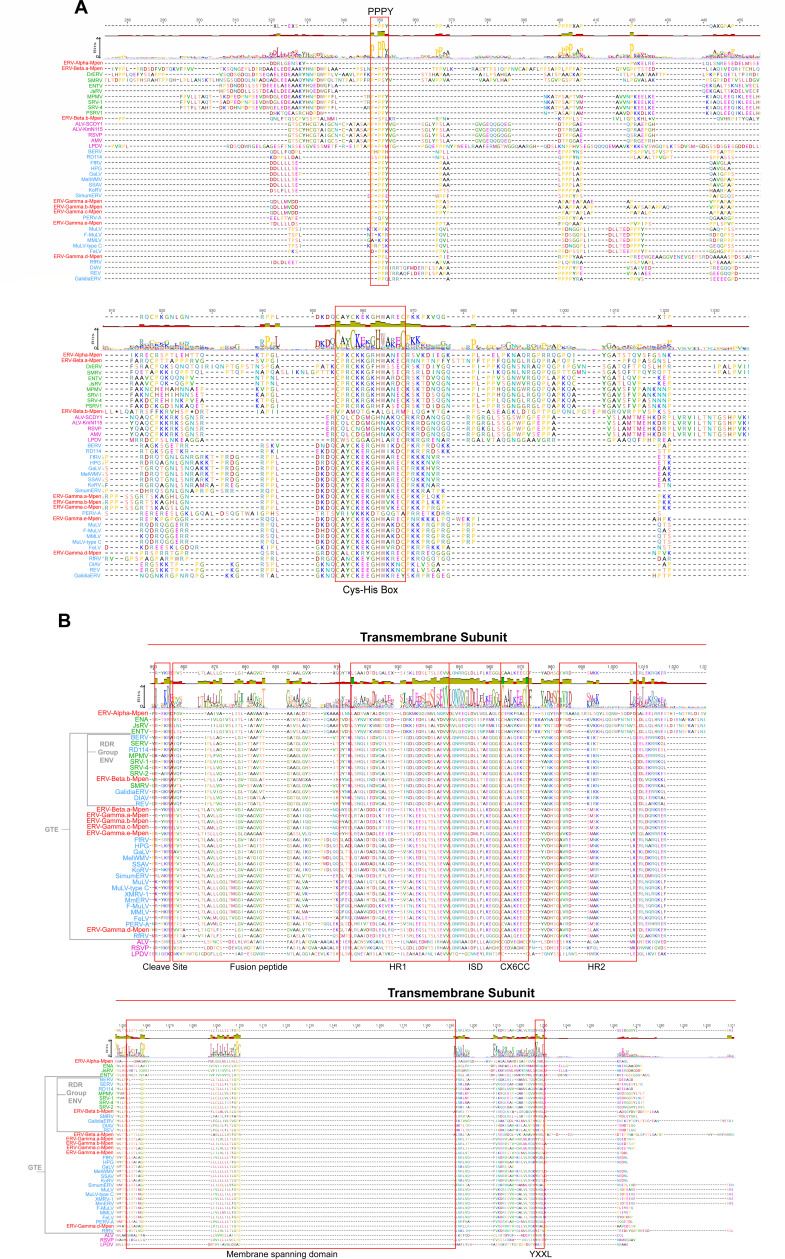
Comparison of MPERVs with GAG and ENV proteins from other endogenous or exogenous retroviruses. (**A**) Alignment of the PPPY motif and Cys-His box regions in GAG proteins between MPERVs and other retroviruses. (**B**) Alignment of transmembrane (TM) unit regions in ENV proteins between MPERVs and other retroviruses. Gamma retroviruses are indicated in blue text, beta retroviruses in green text, and alpha retroviruses in purple text. Sequences identified in this study are highlighted in red text. The hosts of various viruses are shown in Fig. 4 below.

Further characterization of genomic features revealed that all MPERVs, except ERV-Alpha-Mpen and ERV-Beta.b-Mpen (which carries a defective GAG protein), contain the conserved Cys-His box (CX₂CX₄HX₄C) required for capsid formation and the PPPY motif essential for viral particle assembly (3). For the ENV proteins, the transmembrane (TM) subunit harbored cleavage sites (K/R-X-K/R-R) recognized by host cellular furin proteases. Additionally, fusion peptides overlapping with the cleavage sites were identified, critical for processing the envelope protein into two subunits. Downstream of the fusion peptide, heptad repeat regions (HR1 and HR2) were detected, along with a highly conserved immunosuppressive domain (ISD) and CX₆CC motif between HR1 and HR2, both characteristic features for classifying gammaretroviral Env proteins. The cytoplasmic tail region contained the YXXL motif, essential for Env incorporation into virions (14). The membrane-spanning domain (MSD) was also annotated (Fig. 3). The GAG protein of ERV-Beta.a-Mpen contained the major homology region (QGxxExxxxFxx) and two zinc finger domains, consistent with previous findings in other retroviruses (15) (Fig. S1A and B).

Notably, the ENV proteins of ERV-Beta.a-Mpen and ERV-Beta.b-Mpen exhibited an ISD motif (Fig. 3B) and CX₆CC instead of CX₆C. These features collectively indicate that both viruses carry a gamma-type envelope (GTE). The ISD motif is critical for evading innate and adaptive host immunity (16–18) and is absent in classical betaretroviruses and lentiviruses but emerges in recombinant betaretroviruses carrying GTE (19, 20). Collectively, MPERVs share multiple functional motifs with classical ERVs, particularly within structural proteins, underscoring the conservation of core retroviral genes across extended evolutionary timescales.

### Evolution history of MPERVs

**Fig 4:**
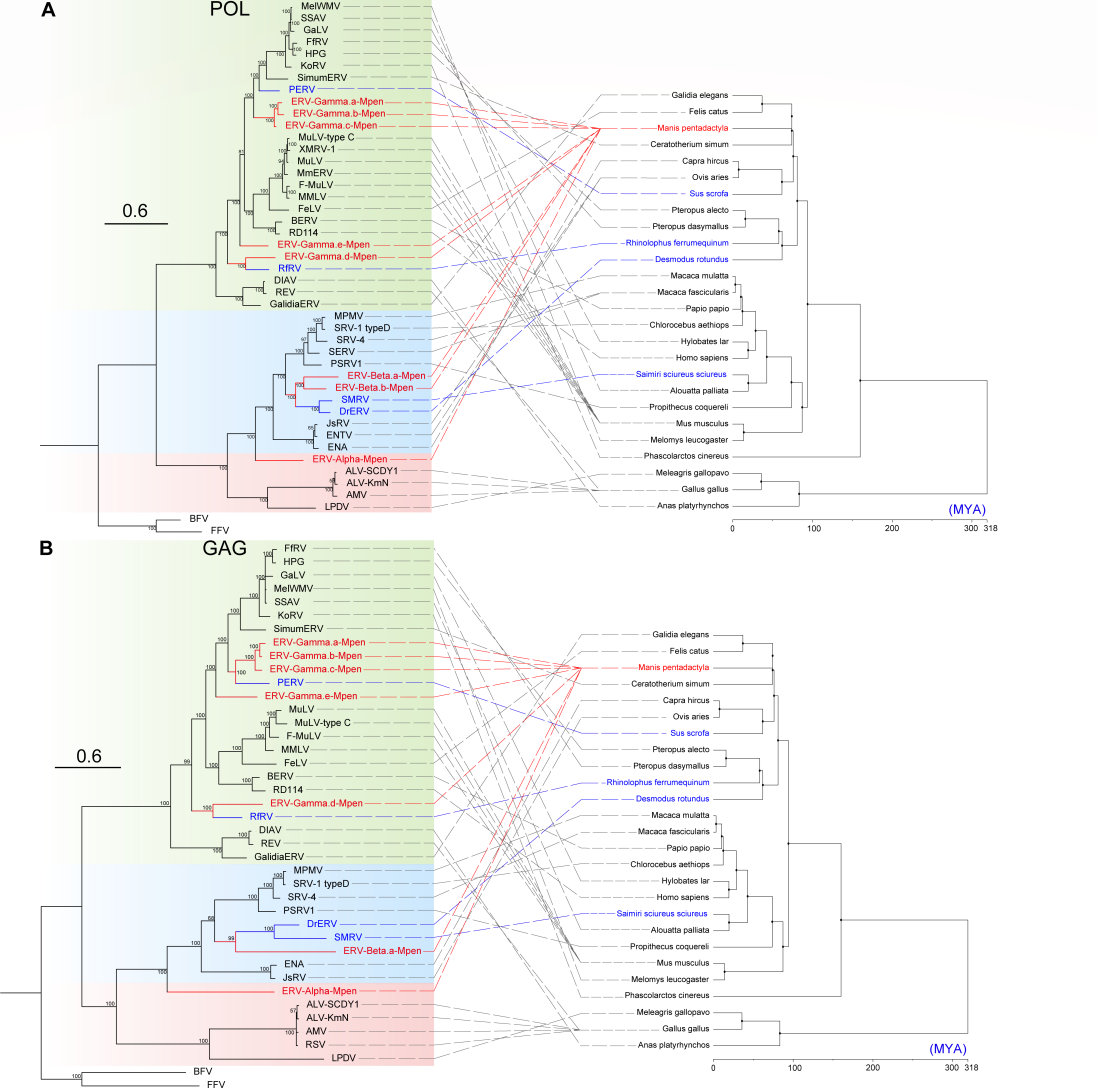
Phylogenetic trees of ERVs and their hosts. The trees are inferred using the amino acid sequences of POL (**A**) and GAG (**B**). The phylogenetic trees of hosts were generated by using TimeTree (http://timetree.org/). Both trees are rooted by foamy retroviruses. The scale bar indicates the number of amino acid changes per site. The newly discovered MPERVs and *Manis pentadactyla* are marked in red text. The phylogenetically related retroviruses and their hosts are marked in blue text.

To elucidate the evolutionary relationships between the newly identified MPERVs and exogenous/endogenous retroviruses, we constructed phylogenetic trees based on long GAG (>500 aa in length), POL (>600 aa in length), and ENV (>400 aa in length) protein sequences (Fig. 4; Fig. S2). In the phylogenetic trees of POL, GAG, and ENV, ERV-Gamma.(a, b, c)-Mpen formed a distinct clade with strong support (PP = 100), suggesting they originated from independent endogenization events of exogenous retroviruses—consistent with previous nucleotide sequence similarity analyses. ERV-Gamma.b-Mpen was excluded from the GAG tree due to a defective GAG protein, while the inconsistent topological positions of ERV-Gamma.d-Mpen in the GAG and POL trees complicate its evolutionary classification. In both the POL and GAG trees, ERV-Gamma-Mpen clustered with porcine endogenous retroviruses, indicating a shared ancestral origin. Additionally, the short branch lengths and the stable clustering of the high-copy ERV-Gamma (a, b, c)-Mpen elements suggest recent and active proliferation of these retroviruses within the Chinese pangolin genome.

The ERV-Beta-Mpen family (ERV-Beta.a-Mpen and ERV-Beta.b-Mpen) clusters within the *Betaretrovirus* genus in the GAG and POL trees, showing close evolutionary ties to retroviruses from the common vampire bat (*Desmodus rotundus*, DrERV) and squirrel monkey (*Saimiri*, SMRV) (Fig. 4). However, the phylogenetic analysis of the ENV region revealed a critical divergence: notably, the ENV protein of ERV-Beta.b-Mpen clustered robustly with those of the RDR interference clade (Fig. S2). This phylogenetic placement aligns with our structural characterization: ERV-Beta.b-Mpen ENV possesses the ISD motif and CX₆CC feature characteristic of this clade, and its TM subunit exhibits high sequence similarity to RDR interference group members (Fig. 3B). Critically, however, further analysis identified a key deficiency: the surface (SU) subunit of ERV-Beta.b-Mpen completely lacks the highly conserved SDGGGXXDXXR receptor-binding motif diagnostic of functional RDR interference ENV (Fig. S1C). This motif is established as essential for binding host cell receptors ASCT1/2 and mediating viral entry (21).

To further assess its evolutionary status, given the low copy number (*n* = 1-4) and the high mutation rate of the ERV-Beta.b-Mpen lineage, we compared the nucleotide sequences of individual insertion loci—specifically ERV-Beta.b2-Mpen and ERV-Beta.b3-Mpen—to those of the RDR interference clade ENV. Notably, certain loci retain the conserved SDGGGXXDXXR receptor-binding motif at the nucleotide level (Fig. S3). Combined with the phylogenetic clustering of the ENV protein and nucleotide-based trees (Fig. S2 and S4C), this definitively establishes the ERV-Beta.b-Mpen lineage as endogenized members of the RDR interference group. However, premature termination codons disrupt the reading frame in these sequences, preventing the translation of a full-length functional ENV protein and indicating profound functional degradation. Although ERV-Beta.b-Mpen retains core structural hallmarks characteristic of the RDR clade ENV, these elements lack the capacity to generate a complete, functional receptor-binding domain. Therefore, we infer that this lineage has lost the ability to interact with ASCT1/ASCT2 receptors during its evolutionary trajectory, resulting in the loss of infectibility and constituting degenerate endogenous members of the RDR interference group.

**Fig 5:**
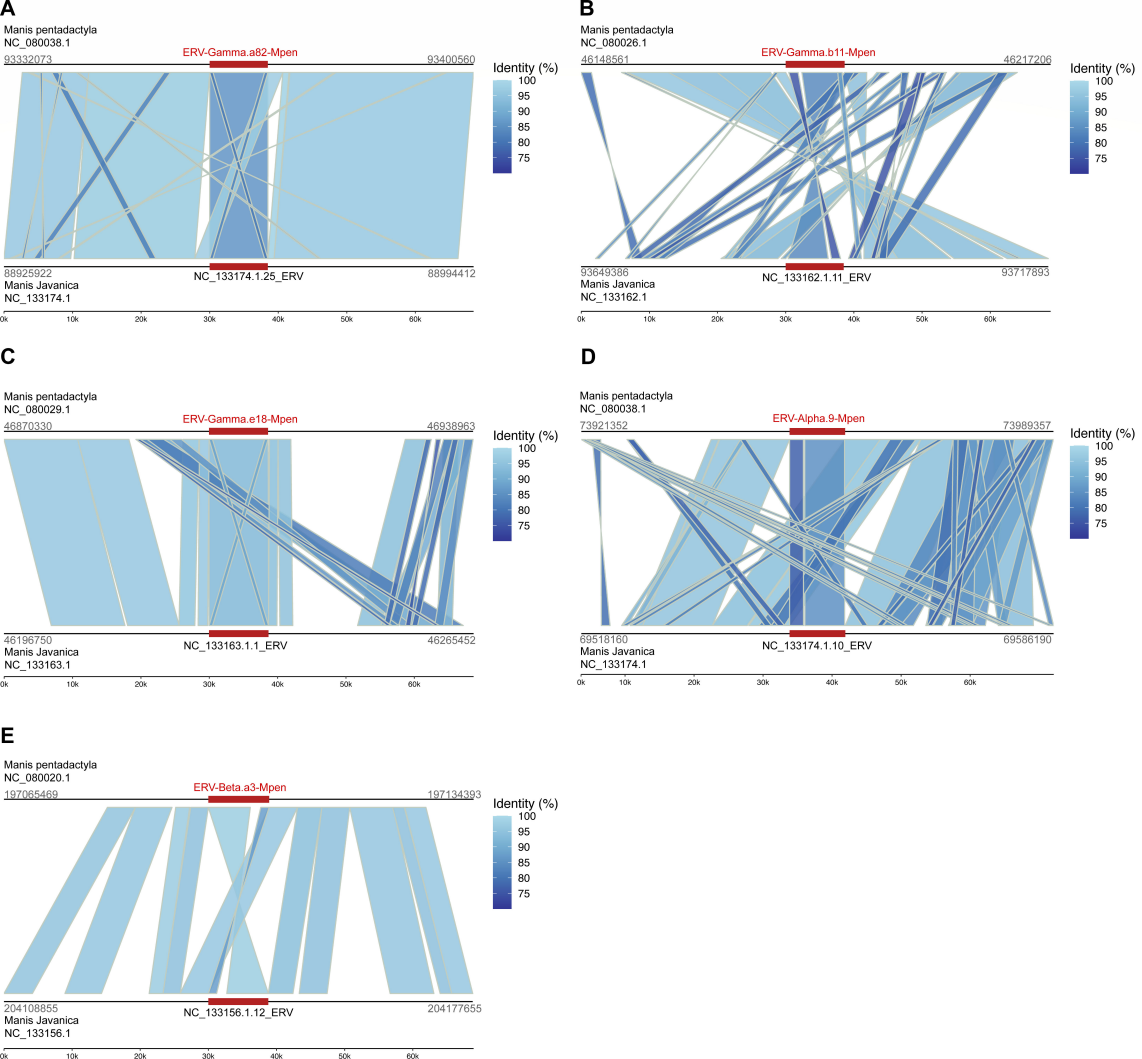
Visualization of orthologous ERV insertions in Chinese pangolin (*Manis pentadactyl*a) and Malayan pangolin (*Manis javanica*). The ERV-associated loci of MPERVs in the Malayan pangolin genome were identified via genome-wide BLASTn, followed by extension of 30,000 bp upstream and downstream for each locus. Pairwise dc-megablast was then performed. The upper tracks represent the homologous insertion coordinates for the ERV-Gamma-Mpen family (**A–C**), ERV-Alpha-Mpen family (**D**), and ERV-Beta-Mpen family (**E**), while the lower tracks represent the homologous insertion coordinates in the Malayan pangolin. The ERV insertion sites are marked with dark red boxes, and the connecting lines indicate homologous regions.

To assess MPERV’s evolutionary context, we used its RT consensus sequence to BLASTn-search mammalian genomes in the National Center for Biotechnology Information (NCBI) database (details in Methods). Significant hits were identified in the Malayan pangolin (*Manis javanica*; assembly GCF_040802235.1). These hits were extended using LTR_Harvest and LTR_finder to identify ERVs with paired LTRs. Analysis of 30,000 bp flanking sequences surrounding these ERVs revealed five homologous insertion events (Fig. 5A-5E), suggesting the possibility of the vertical transmission of MPERVs between the Malayan and Chinese pangolins, rather than MPERV being a Chinese pangolin-specific ERV.

### Phylogenetic analysis of paired LTRs and SoloLTRs in MPERVs

To investigate the evolutionary history of MPERV-associated LTRs, we identified solitary LTRs (soloLTRs) in the Chinese pangolin (*Manis pentadactyla*) genome. Following the previous nomenclature, we designated them as: SoloLTR-Alpha.n-Mpen (*n* = 19), SoloLTR-Beta.an-Mpen (*n* = 8), SoloLTR-Beta.bn-Mpen (*n* = 34), SoloLTR-Gamma.an-Mpen (*n* = 119), SoloLTR-Gamma.bn-Mpen (*n* = 180), SoloLTR-Gamma.cn-Mpen (*n* = 185), SoloLTR-Gamma.dn-Mpen (*n* = 163), and SoloLTR-Gamma.en-Mpen (*n* = 128). Additionally, consensus sequences for each lineage were constructed based on nucleotide sequence similarity and labeled with Roman numerals.

Phylogenetic analysis was performed on the paired LTRs of previously identified MPERV-unique insertion sites, including solo LTRs (Fig. S5 and S6). The results revealed that the paired LTRs of ERV-Gamma.a-Mpen and ERV-Gamma.b-Mpen exhibited partial nesting in the phylogenetic tree topology (Fig. S6). A similar pattern was observed between ERV-Gamma.c-Mpen and ERV-Gamma.e-Mpen, suggesting a shared ancestral origin or a close evolutionary relationship of their progenitor viruses prior to endogenization. All other families formed distinct monophyletic clades.

**Fig 6:**
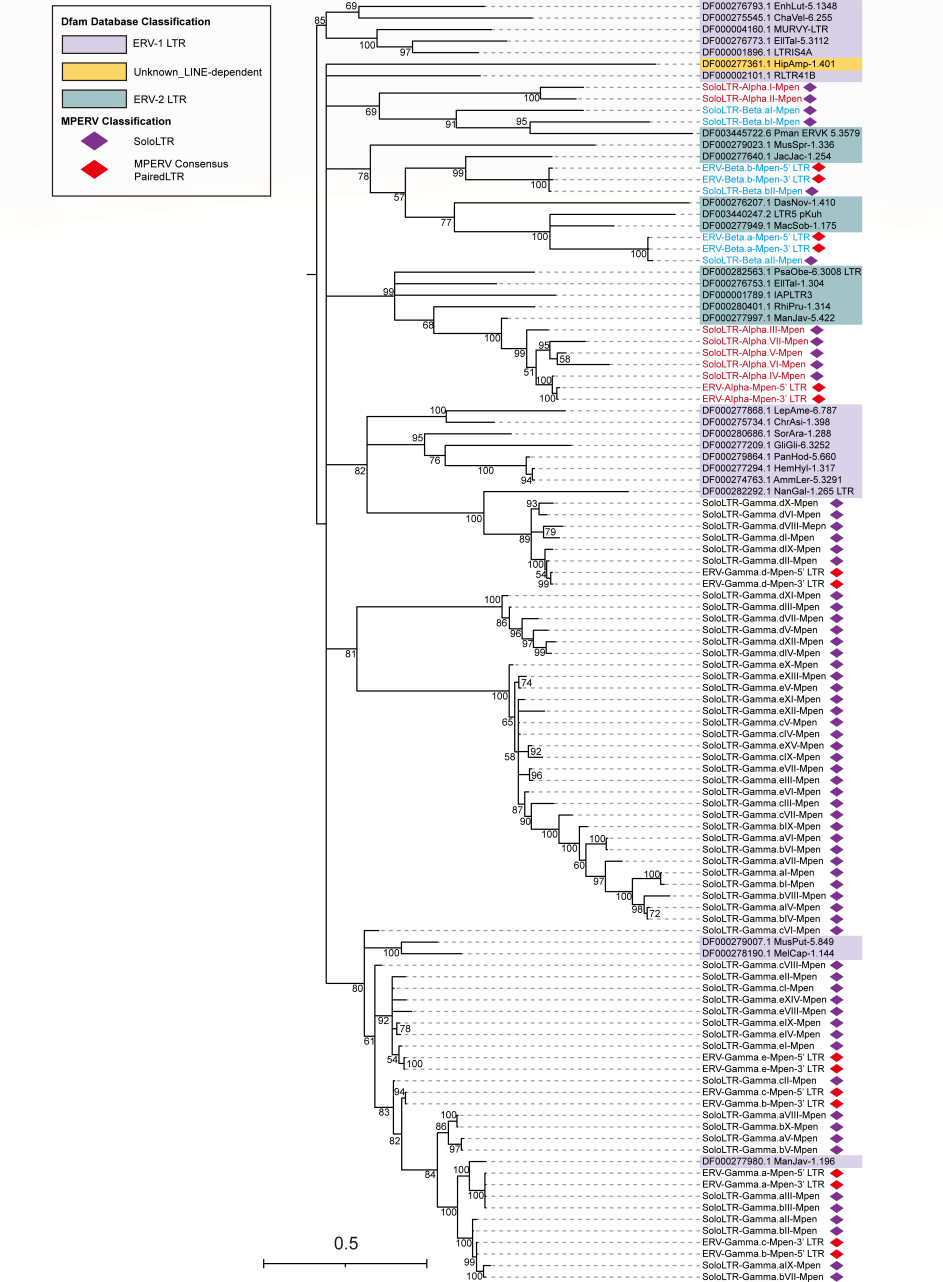
Phylogenetic tree of the MPERV-associated paired LTRs, solo LTRs, and LTRs from the Dfam database. The phylogenetic tree was constructed using Bayesian inference, with posterior probabilities supporting each node indicated numerically. The scale bar represents the nucleotide substitutions per site. Members from distinct family lineages are color-coded as follows: Alpha-family MPERVs (red), Beta-family MPERVs (blue), and Gamma-family MPERVs (black). The Dfam database classifications are highlighted with distinct background colors. Red diamonds denote paired MPERV LTRs extracted from consensus sequences, while purple diamonds represent solo LTRs. Reference sequence names include their corresponding Dfam database accession numbers.

To contextualize MPERV LTRs within a broader evolutionary framework, we conducted phylogenetic comparisons with LTRs annotated as long terminal repeat elements in the Dfam database (22) (Fig. 6). LTRs from the Alpha and Beta families clustered with the Dfam ERV2 group, while those from the Gamma family aligned with the Dfam ERV1 group. However, due to accumulated mutations over evolutionary time, the individual lineages within the Gamma family did not form well-separated clades. Notably, some solo LTRs and paired LTRs from the MPERV Alpha and Gamma families clustered closely with LTRs derived from *Manis javanica* (DF000277997.1 ManJav-5.422 and DF000277980.1 ManJav-1.196), further supporting the possibility of vertical transmission of MPERV between these two pangolin species.

### Estimation of insertion times for MPERVs

Given the complex evolutionary processes revealed by the phylogenetic analyses of MPERVs, we estimated the insertion times of different MPERV lineages using a divergence-based dating method for paired LTRs (23). This approach aimed to further elucidate the evolutionary history of MPERVs. To ensure accuracy, only MPERV elements with intact viral genomes and complete LTR sequences were included in the analysis (Table S1). Our results indicate that the insertion times for the ERV-Gamma-Mpen lineage range from 0 to 18.38 million years ago (Mya), while the ERV-Beta-Mpen lineage exhibits insertion times spanning 0 to 14.29 Mya, and the ERV-Alpha-Mpen lineage dates between 1.41 and 3.47 Mya. These divergence times are close to the divergence time between *Manis pentadactyla* (Chinese pangolin) and *Manis javanica* (Malayan pangolin) (~16.40 Mya). Given the lack of pangolin-specific neutral nucleotide substitution rates and the potential error introduced by using the average mammalian neutral substitution rate, it can be inferred that MPERVs integrated into the genome of a more ancient common ancestor of *M. pentadactyla* and *M. javanica*.

## DISCUSSION

This study conducted an analysis of a subset of endogenous retroviruses (ERVs) in the genome of the Chinese pangolin (*Manis pentadactyla*), identifying three novel genera (Alpha-, Beta-, and Gamma-MPERVs) and elucidating their complex evolutionary trajectories. According to the International Committee on Taxonomy of Viruses (ICTV) classification, exogenous retroviruses are categorized into seven genera: *Alpharetrovirus*, *Betaretrovirus*, *Gammaretrovirus*, *Deltaretrovirus*, *Epsilonretrovirus*, *Lentiretrovirus*, and *Spumaretrovirus*. However, endogenous retroviruses do not strictly adhere to this classification system (24). The newly identified pangolin ERVs in this study also follow this trend.

Based on their relationship with exogenous retroviruses, ERVs are generally classified into three groups: (i) ERVs related to *Gammaretrovirus* and *Epsilonretroviruses*; (ii) ERVs related to *Alpharetrovirus* and *Betaretroviruses*; and (iii) ERVs related to *Spumaretrovirus*. For instance, the identified ERV-Alpha-Mpen sequences exhibit phylogenetic positions between α- and β-retroviruses in the GAG, POL, and ENV protein phylogenies. Using the exogenous retrovirus classification system, the classification of ERV-Alpha-Mpen remains ambiguous. However, in the phylogenetic tree based on reverse transcriptase (RT) sequences, ERV-Alpha-Mpen clusters with *Alpharetrovirus*. While previous studies have established that almost all *Alpharetrovirus* hosts are avian species (25), this finding suggests a potential expansion of the *Alpharetrovirus* host range. For now, ERV-Alpha-Mpen is tentatively categorized as a Group II ERV. Based on its clustering patterns in the GAG, POL, and ENV phylogenies, we hypothesize that ERV-Alpha-Mpen may have originated from an exogenous retrovirus that has since become extinct prior to its endogenization.

The evolutionary patterns of betaretroviruses are also intriguing. The length of their LTRs (387 nt) is shorter than that of typical betaretroviruses, which generally exceed 1,000 nt (26). Previous studies have shown that betaretroviruses generally exhibit two types of *env* proteins: β-type *env* and γ-type *env*. In our findings, the newly identified ERV-Beta.a-Mpen displays evidence of recombination, a process thought to facilitate cross-species transmission (27). Previous reports have suggested that the replacement of β-type Env with γ-type Env can facilitate cross-species jumps among vertebrates. Such an event may have occurred in the case of *Python molurus* ERV (PyERV), which has been identified in the genomes of two python species (28). The *gag-pol* region of PyERV clusters with Class II ERVs and is most closely related to betaretroviruses, while its ENV gene exhibits characteristics typical of murine γ-retroviruses. In our study, a highly similar scenario was observed concerning two lineages of betavirus MPERVs (ERV-Beta.a-Mpen and ERV-Beta.b-Mpen). The GAG-POL regions of these two distinct Chinese pangolin betavirus lineages exhibit clear betaretroviral features. The ENV protein of ERV-Beta.b-Mpen shows high homology to gamma-type ENV from the RDR interference group; however, it harbors a premature termination codon across nearly the entire SU region. Nevertheless, the nucleotide sequence analysis revealed that ERV-Beta.b-Mpen possesses the characteristic SDGGGXXDXXR motif of the RDR interference group, indicating that this lineage is a novel endogenous member of this group. This aligns with previous findings that the SU subunit, being the most exposed part to the host immune system, is subject to strong adaptive pressures (29). In contrast, ERV-Beta.a-Mpen presents a different picture—while its GAG-POL region also belongs to betaretroviruses, its ENV SU and TM domains display higher homology to gamma-type endogenous retroviruses within the pangolin genome (Fig. 3 and 4). We hypothesize that an ENV-swapping recombination event also occurred in the Chinese pangolin genome, enabling beta-family MPERVs to colonize the host genome by acquiring a gamma-type ENV. Furthermore, we speculate that the observed variation in lineage copy numbers between ERV-Beta.a-Mpen and ERV-Beta.b-Mpen, coupled with the former possessing a more intact ENV protein, may be attributable to their distinct recombinant ENV strategies.

## MATERIALS AND METHODS

### Genome screening and identification of MPERVs

To identify potential endogenous retrovirus (ERV) elements within the Chinese pangolin genome, we performed searches using Diamond BLASTx (v0.9.24) (30) and tBLASTn (31) against the genome, with POL protein sequences from all known retroviruses serving as queries (Table S4). Significant hits were filtered using a threshold of 30% sequence identity covering at least 40% of the region and an E-value cutoff of 1E−5. A phylogenetic analysis was then conducted to confirm whether the identified sequences represented *bona fide* ERVs based on their clustering with representative retroviruses in well-supported clades (posterior probability > 70). Additionally, we employed LTR_FINDER (32) and LTR_harvest (33) to extend the significant matches, identifying full-length long terminal repeats (LTRs) and annotating key features, such as the primer binding site (PBS), polypurine tract (PPT), and target site duplications (TSDs). LTR_FINDER parameters: “-w 2 -C -D 15000 -d 1000 -L 7000 -l 100 -p 20 -M 0.85” and LTR_harvest parameters: “-tis -suf -lcp -des -ssp -sds -dna” for gt suffixerator and default settings were applied for LTR filtering. The coordinates of all MPERVs can be found in Table S1.

### Consensus genome construction and annotation

Full-length ERVs were used as queries to search for ERV copies using BLASTn. The hit parts of sequences longer than 4 kb with 80% identity were regarded as copies of each lineage. Consensus sequences for each MPERV were generated using Geneious Prime (v2019.0), requiring nucleotide similarity greater than 90%. Open reading frames (ORFs) were identified using the ORFfinder tool from the NCBI database (https://www.ncbi.nlm.nih.gov/orffinder/) and validated through a BLASTp analysis (31). Conserved domains within the sequences were annotated using the CDD search tool (https://www.ncbi.nlm.nih.gov/cdd/) (34).

### Phylogenetic analysis

Protein sequences of interest were aligned using the MUSCLE algorithm implemented in MEGA (v10.1.8) under default settings (35). Phylogenetic trees were constructed using Bayesian inference in MrBayes v3.2.7 (36). For this analysis, amino acid substitution models were evaluated under the mixed model framework (“prset aamodelpr = mixed”), which allows selection among 10 built-in models. When building nucleotide phylogenetic trees, using “nst = 6 and rates = invgamma,” the maximum number of generations was set to 10 million, with analyses terminating when the standard deviation of split frequencies was below 0.01. Phylogenetic trees were visualized and edited using FigTree v1.4.4 (http://tree.bio.ed.ac.uk/software/figtree/) and Adobe Illustrator 2020 (v26.0.1). For all paired LTRs and solo LTRs associated with MPEREVs in Fig. 6, multiple sequence alignment was performed using MAFFT(37) (L-INS-I algorithm), followed by trimming with TrimAI’s automatic algorithm (38) to remove poorly aligned regions. Phylogenetic trees were constructed using IQ-TREE (v1.6.12) based on a large data set of full-length viral LTR pairs. Maximum likelihood (ML) analysis was employed with the "-MFP" parameter to automatically select the best-fit substitution model (TPM2 +F + R4). Node support was assessed using 100 bootstrap replicates (39).

### Evolutionary context and homology identification of MPERVs

To contextualize the MPERVs identified in this study within a broader evolutionary framework, we used all RT sequences derived from MPERV consensus sequences as queries to perform BLASTn searches (default parameters) against the NCBI genome database (https://www.ncbi.nlm.nih.gov/datasets/genome/), with filters restricted to: (i) mammalian species, (ii) reference or annotated genomes, and (iii) chromosome-level or complete genome assemblies. This yielded a final data set of 159 qualified genomes, yet significant hits were exclusively detected in the Malayan pangolin (*Manis javanica*; assembly accession: GCF_040802235.1). A threshold of ≥80% sequence similarity covering ≥80% of the query region was applied. Further extension and analysis of flanking sequences using LTR_harvest and LTR_finder confirmed the presence of characteristic paired LTRs. All identified Malayan pangolin ERV RT loci corresponding to MPERV RT sequences, along with complete ERV annotations, were systematically compiled in Table S3.

To assess the potential vertical transmission between MPERVs and their Malayan pangolin counterparts, we first extracted 30,000 bp flanking sequences surrounding ERV insertion sites. Homologous regions were then detected using dc-megablast (E-value ≤ 1E−5). Pairwise comparisons were conducted to evaluate vertical inheritance based on two criteria: (i) nucleic acid similarity ≥ 85% between ERV insertion regions, and (ii) ≥50% coverage of flanking sequence alignments. Alignments shorter than 500 bp were excluded to avoid spurious hits from short repeats. The dc-megablast results were visualized using gggenomes (https://github.com/thackl/gggenomes).

### Identification of solo LTRs

To identify solo LTRs in the Chinese pangolin, we performed BLASTn searches using LTR sequences from MPERVs as queries. Filtering of significant hits was performed using regions with E-values of 1E−5, requiring >10% coverage and >80% sequence identity. Hits without paired LTRs and MPERV remnants near 15,000 bp were classified as soloLTRs. Sequences showing >90% similarity were considered copies of the same soloLTR. Consensus sequences were generated using Geneious Prime and included in the phylogenetic tree analysis. The coordinates of the soloLTR independent copies identified in this study and the consensus sequences of each lineage can be found in the Supplementary material.

### Molecular dating of MPERVs

The insertion times of MPERVs were estimated using the formula *T=(D/R)/2*, where *T* represents the integration time (in million years, Mya); *D* is the nucleotide divergence between paired LTRs (Kimura 2-parameter model); and *R* is the neutral substitution rate per site per year. As no species-specific substitution rate is available for the Chinese pangolin, we adopted the average neutral substitution rate for mammals, 2.2 × 10^−9^ substitutions per site per year, for our calculations (40).

## Data Availability

The genome assemblies of the Chinese pangolin are available at the National Center for Biotechnology Information (NCBI, https://www.ncbi.nlm.nih.gov/genome/), and the accession number is GCF_030020395.1. Additional data, figures, and datasetsdata sets needed to support the conclusions detailed in the article are included in the Supplemental material. The multiple sequence alignment files of the phylogenetic trees used in this study, along with the consensus sequences of MPERVs and soloLTRs, are available at: https://github.com/Tczhang0606/Additional-Files.

## SUPPLEMENTAL MATERIAL

Supplemental figures spectrum.03203-24-s0001.pdf Figures S1 to S6.

Table S1 spectrum.03203-24-s0002.xlsx Genomic loci, Dfam classification, estimated integration events, and pairwise LTR divergence of MPERVs.

Table S2 spectrum.03203-24-s0003.xlsx Genomic coordinates of the MPERVs soloLTRs, Dfam database classification, associated MPERV families, and lineage information for each individual soloLTR.

Table S3 spectrum.03203-24-s0004.xlsx Genomic mapping of the RT sequences associated with MPERVs in the Malayan pangolin genome.

Table S4 spectrum.03203-24-s0005.xlsx Genomic mapping of the ERV insertion sites with the paired LTRs associated with MPERVs in the Malayan pangolin genome.

Table S5 spectrum.03203-24-s0006.xlsx Information about the representative retroviruses.

ASM does not own the copyrights to Supplemental Material that may be linked to, or accessed through, an article. The authors have granted ASM a non-exclusive, world-wide license to publish the Supplemental Material files. Please contact the corresponding author directly for reuse.
