# Supplementary material for: Diversity and evolutionary history of endogenous retroviruses in the genome of Manis pentadactyla
Source: Microbiol Spectr. 2025 Sep 30;13(11):e03203-24. doi: 10.1128/spectrum.03203-24 (PMC12584738; doi:10.1128/spectrum.03203-24)
Supplement: Supplemental figures — Figures S1 to S6. [file spectrum.03203-24-s0001.pdf]

Supplementary Materials

Supplementary Materials file includes:

Fig. S1 Comparison of MPERVs GAG and ENV proteins from other endogenous or exogenous retroviruses.

Fig. S2 Phylogenetic trees of MPERVs.

Fig. S3 Nucleotide sequence alignment of ENV regions from ERV-Beta.b-Mpen members and RDR interference groups.

Fig. S4 Nucleotide phylogenetic trees of MPERVs.

Fig. S5 Nucleotide phylogenetic trees of paired long terminal repeats (LTRs) and Solo LTRs from various MPERV families in the Chinese pangolin.

Fig. S6 Phylogenetic tree of MPERV LTRs.

Table. S1 Genomic loci, Dfam classification, estimated integration events, and pairwise LTR divergence of MPERVs.

Table. S2 Genomic coordinates of MPERVs soloLTRs, Dfam database classification, associated MPERVs families, and lineage information for each individual soloLTR.

Table. S3 Genomic mapping of RT sequences associated with MPERVs in the Malayan pangolin genome.

Table. S4 Genomic mapping of ERV insertion sites with paired LTRs associated with MPERVs in the Malayan pangolin genome.

Table. S5 Information about the representative retroviruses.

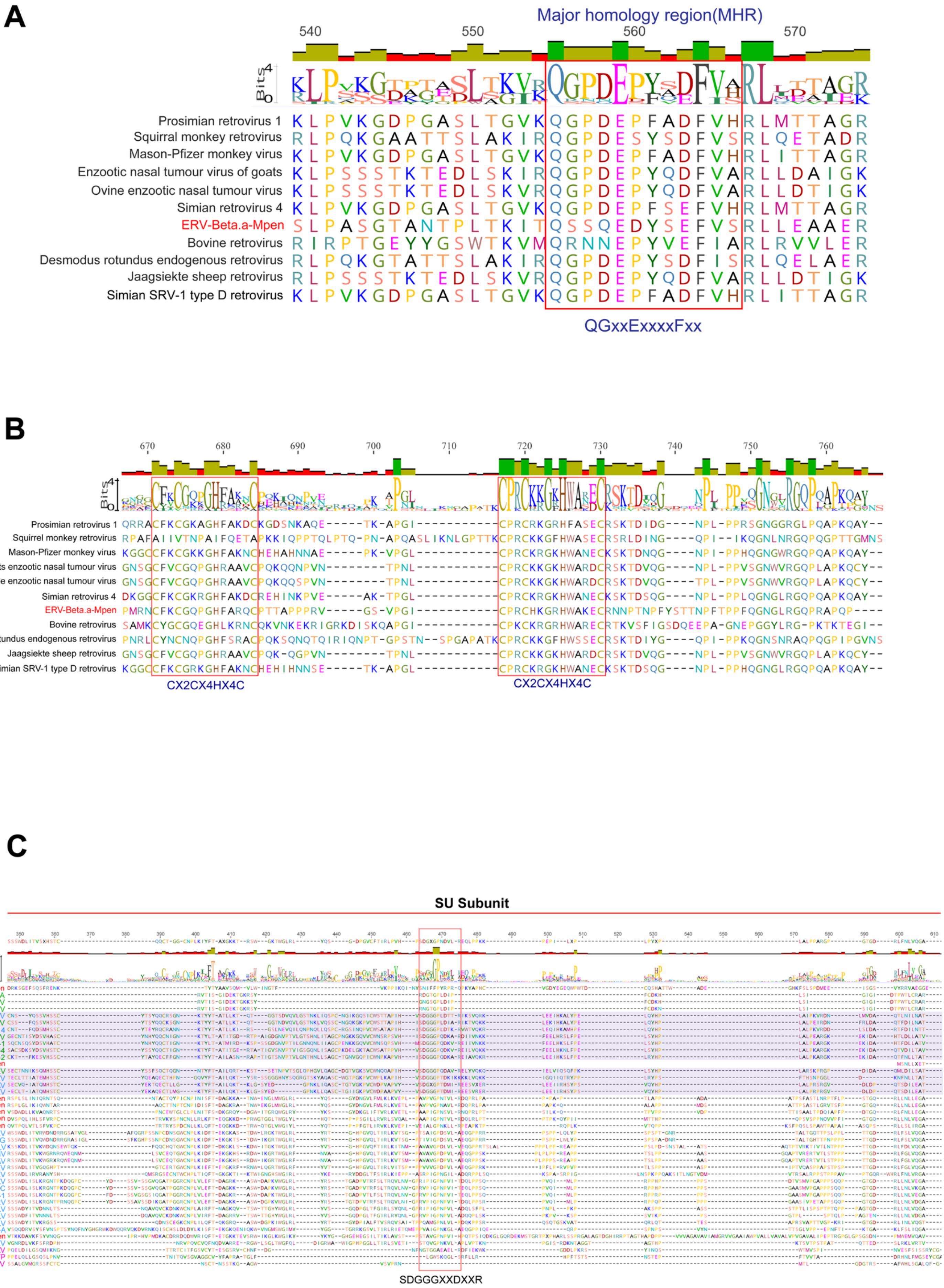

A
Major homology region(MHR)
540
550
560
570
Bits
Prosimian retrovirus 1 KLPVKGDPGASLTGVKQGPDEPFADFVHRLMTTAGR
Squirral monkey retrovirus RLPQKGAATTSLAKIRQGPDESYSDFVSRLQETADR
Mason-Pfizer monkey virus KLPVKGDPGASLTGVKQGPDEPFADFVHRLITTAGR
Enzootic nasal tumour virus of goats KLPSSSTKTEDLSKIRQGPDEPYQDFVARLLDAIGK
Ovine enzootic nasal tumour virus KLPSSSTKTEDLSKVRQGPDEPYQDFVARLLDTIGK
Simian retrovirus 4 KLPVKGDPGASLTGVKQGPDEPFSEFVHRLMTTAGR
ERV-Beta.a-Mpen SLPASGTANTPLTKITQSSQEDYSEFVSRLLEAAER
Bovine retrovirus RIRPTGEYYGSWTKVMQRNNEPYVEFIARLRVVLER
Desmodus rotundus endogenous retrovirus RLPQKGTATTSLAKIRQGPDEPYSDFISRLQELAER
Jaagsiekte sheep retrovirus RLPSSSTKTEDLSKVRQGPDEPYQDFVARLLDTIGK
Simian SRV-1 type D retrovirus KLPVKGDPGASLTGVKQGPDEPFADFVHRLITTAGR
QGxxExxxxFxx
B
Prosimian retrovirus 1
Squirrel monkey retrovirus
Mason-Pfizer monkey virus
Goats enzootic nasal tumour virus
Ovine enzootic nasal tumour virus
Simian retrovirus 4
ERV-Beta.a-Mpen
Bovine retrovirus
Desmodus rotundus endogenous retrovirus
Jaagsiekte sheep retrovirus
Simian SRV-1 type D retrovirus
CX2CX4HX4C
CX2CX4HX4C
C
SU Subunit
ERV-Alpha-Mpen
ERV-Beta.a-Mpen
ERV-Beta.b-Mpen
ERV-Gamma.a-Mpen
ERV-Gamma.b-Mpen
ERV-Gamma.c-Mpen
ERV-Gamma.e-Mpen
ERV-Gamma.d-Mpen
SDGGGXXDXXR

Fig. S1. Comparison of MPERVs GAG and ENV proteins from other endogenous or exogenous retroviruses. (A) Alignment of major homology region (MHR) in GAG proteins between MPERVs (Beta) and other Beta retroviruses. (B) Alignment of zinc finger domains in GAG proteins between MPERVs (Beta) and other Beta retroviruses. (C) Alignment of SU (Surface subunit) regions in ENV proteins between MPERVs and other retroviruses. The newly discovered MPERVs are marked in red text. RDR interference group viruses are shaded in purple.

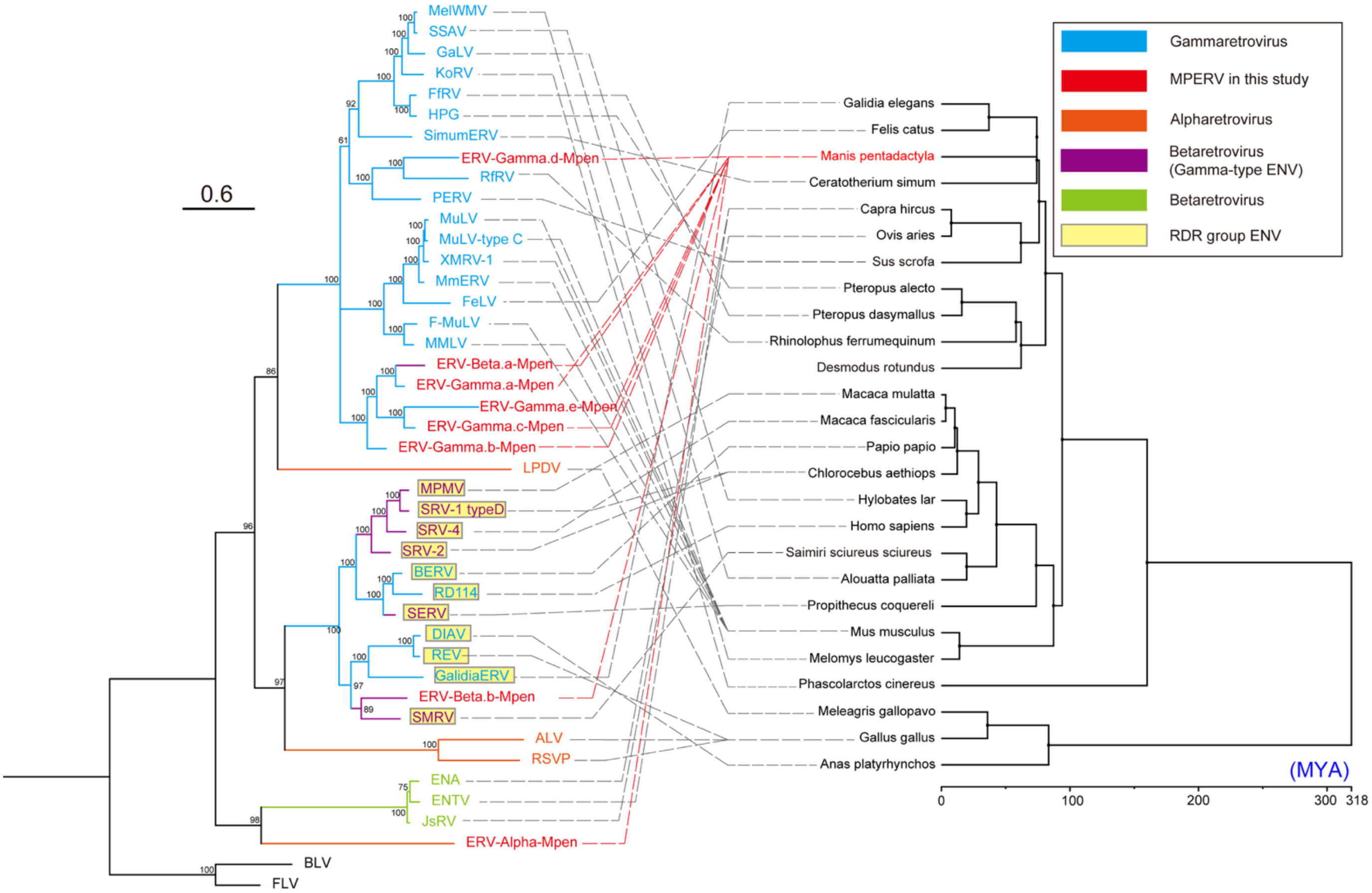

Fig. S2. Phylogenetic trees of MPERVs. The phylogenetic tree was constructed using Bayesian inference based on the consensus amino acid sequences of MPERV ENV proteins. Numbers at nodes represent the posterior probability supporting each node. The scale bar indicates amino acid substitutions per site. ENV proteins belonging to the RDR interference group are highlighted with yellow boxes. MPERVs identified in this study are labeled in red text. Clades containing *Betaretrovirus* carrying *Gammaretrovirus*-type ENV are colored purple.

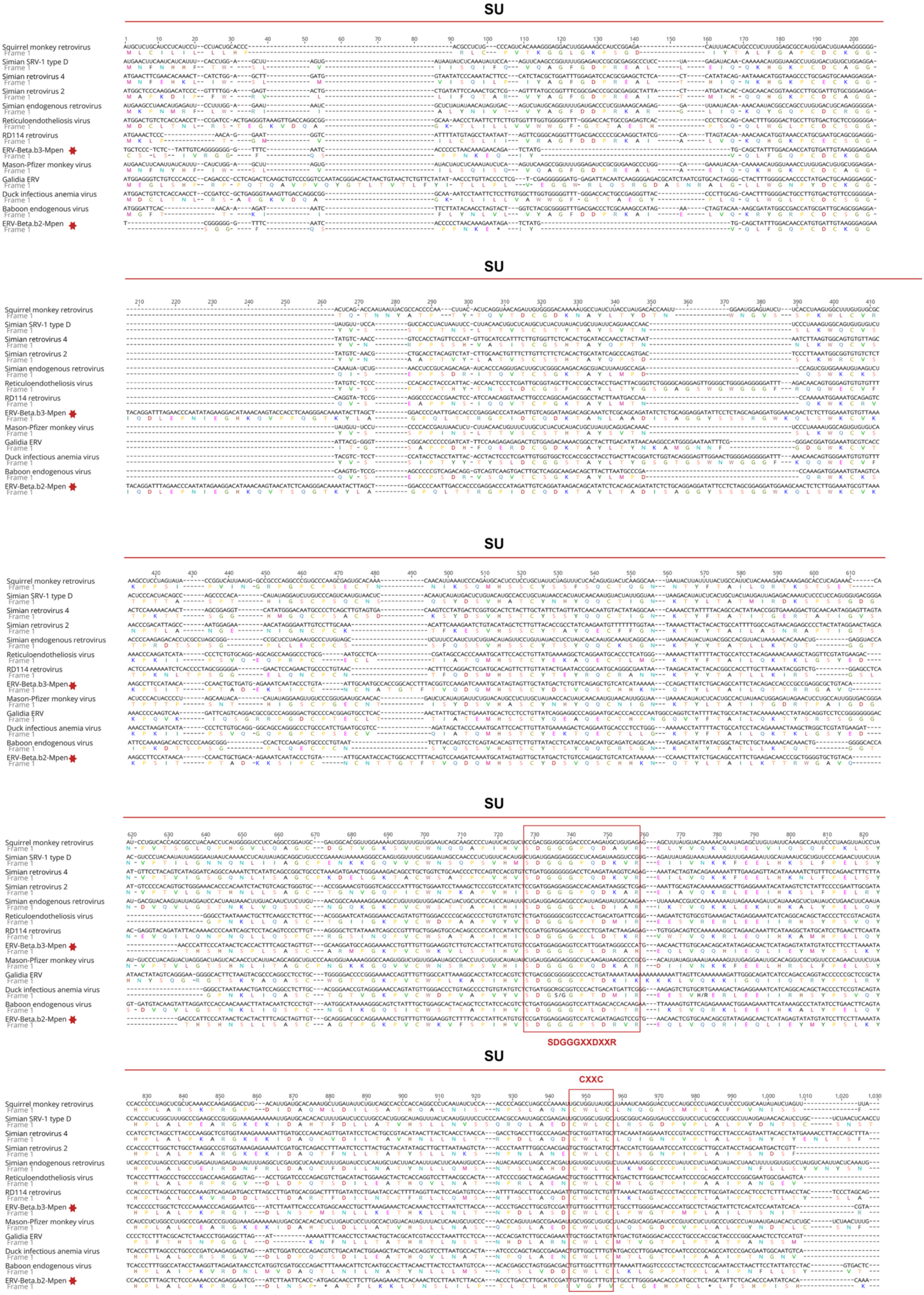
SU
Squirrel monkey retrovirus
Simian SRV-1 type D
Simian retrovirus 4
Simian retrovirus 2
Simian endogenous retrovirus
Reticuloendotheliosis virus
RD114 retrovirus
ERV-Beta.b3-Mpen
Mason-Pfizer monkey virus
Galidia ERV
Duck infectious anemia virus
Baboon endogenous virus
ERV-Beta.b2-Mpen
Frame 1
SDGGGXXDXXR
CXXC

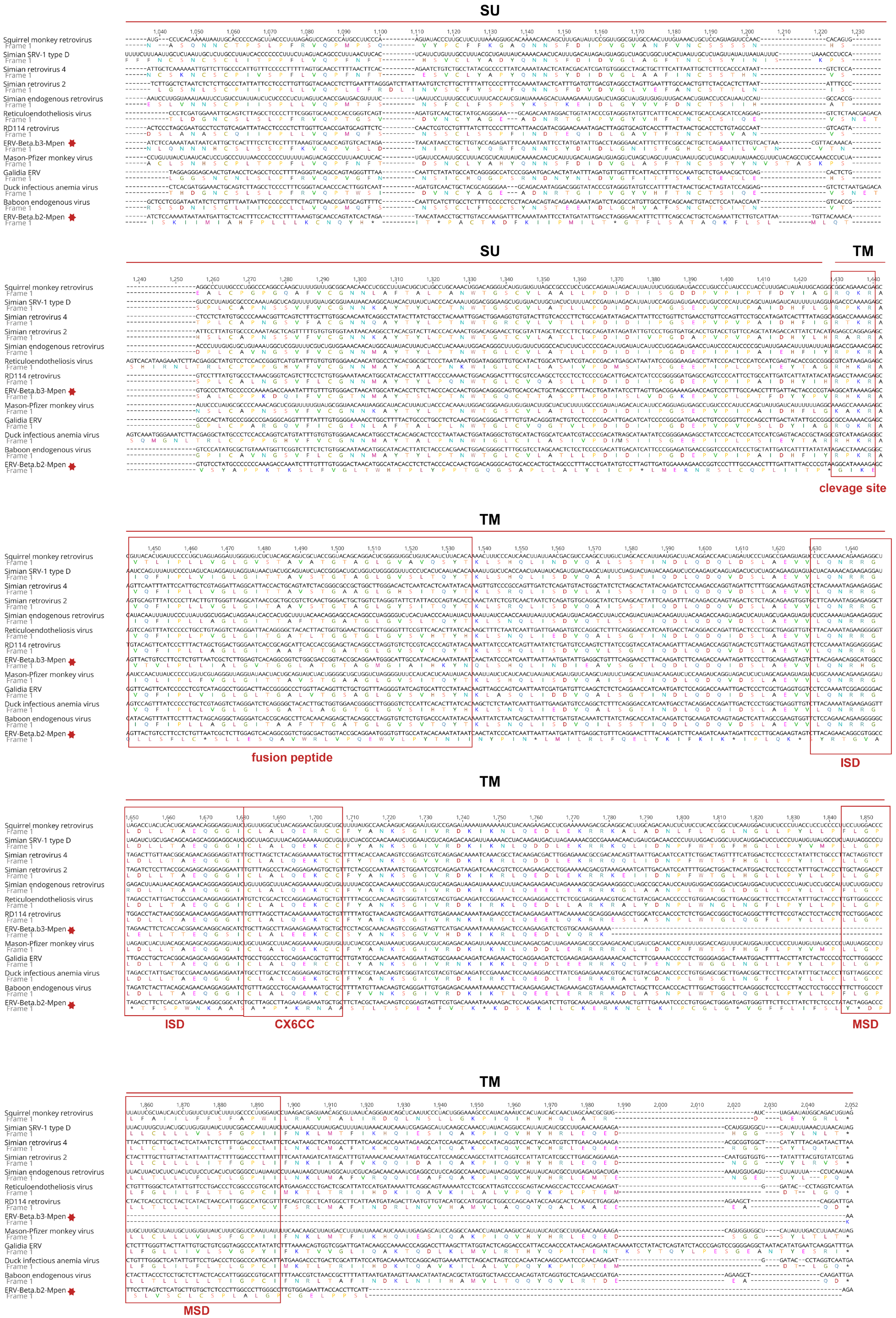

SU
TM
Squirrel monkey retrovirus
Simian SRV-1 type D
Simian retrovirus 4
Simian retrovirus 2
Simian endogenous retrovirus
Reticuloendotheliosis virus
RD114 retrovirus
ERV-Beta.b3-Mpen
Mason-Pfizer monkey virus
Galidia ERV
Duck infectious anemia virus
Baboon endogenous virus
ERV-Beta.b2-Mpen
Frame 1
clevage site
fusion peptide
ISD
CX6CC
MSD

Fig. S3. Nucleotide sequence alignment of ENV regions from ERV-Beta.b-Mpen members and RDR interference groups. Structural domains of ENV, the Surface Unit (SU) and Transmembrane Unit (TM), are demarcated by horizontal lines. Key functional motifs/regions are annotated: the SDGGGXXDXXR motif, CXXC motif, cleavage site, fusion peptide, immunosuppressive domain (ISD), CX6CC motif, and membrane-spanning domain (MSD). Translated amino acids are shown in color. The ERV-Beta.b2-Mpen and ERV-Beta.b3-Mpen sequences identified in the present study are indicated by red asterisks. Other ERV-Beta.b-Mpen family members were excluded from the analysis due to the absence of ENV region nucleotide sequences.

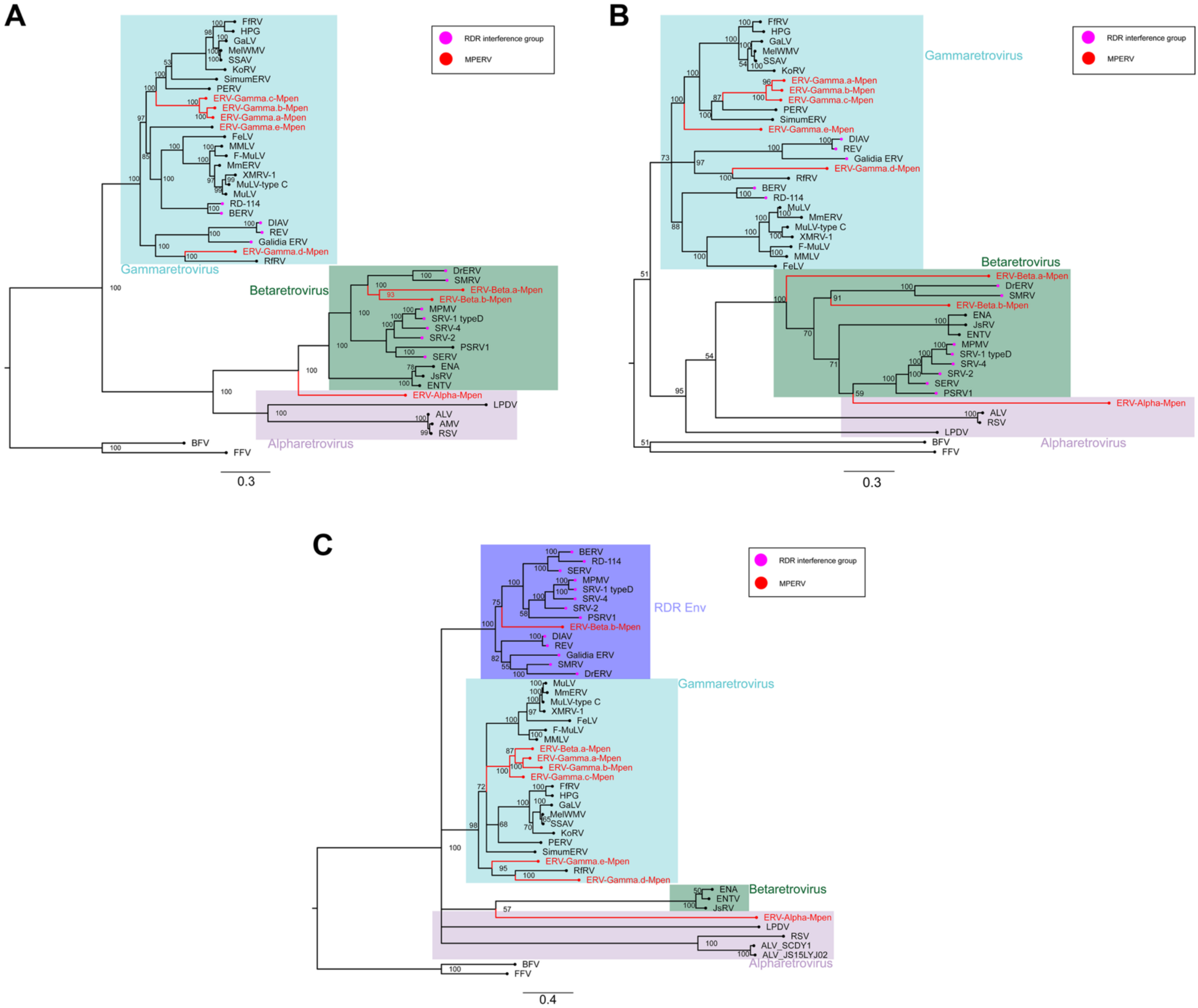

Fig. S4. Nucleotide phylogenetic trees of MPERVs. Phylogenetic trees were constructed based on nucleotide sequences from the (A) POL, (B) GAG, and (C) ENV regions of MPERV consensus sequences using Bayesian inference. All trees are rooted with foamy retroviruses. Newly discovered MPERVs are marked in red text. The scale bar indicates the number of amino acid changes per site. In panel (C), ENV sequences belonging to the RDR interference group are labeled in purple at the branch termini.

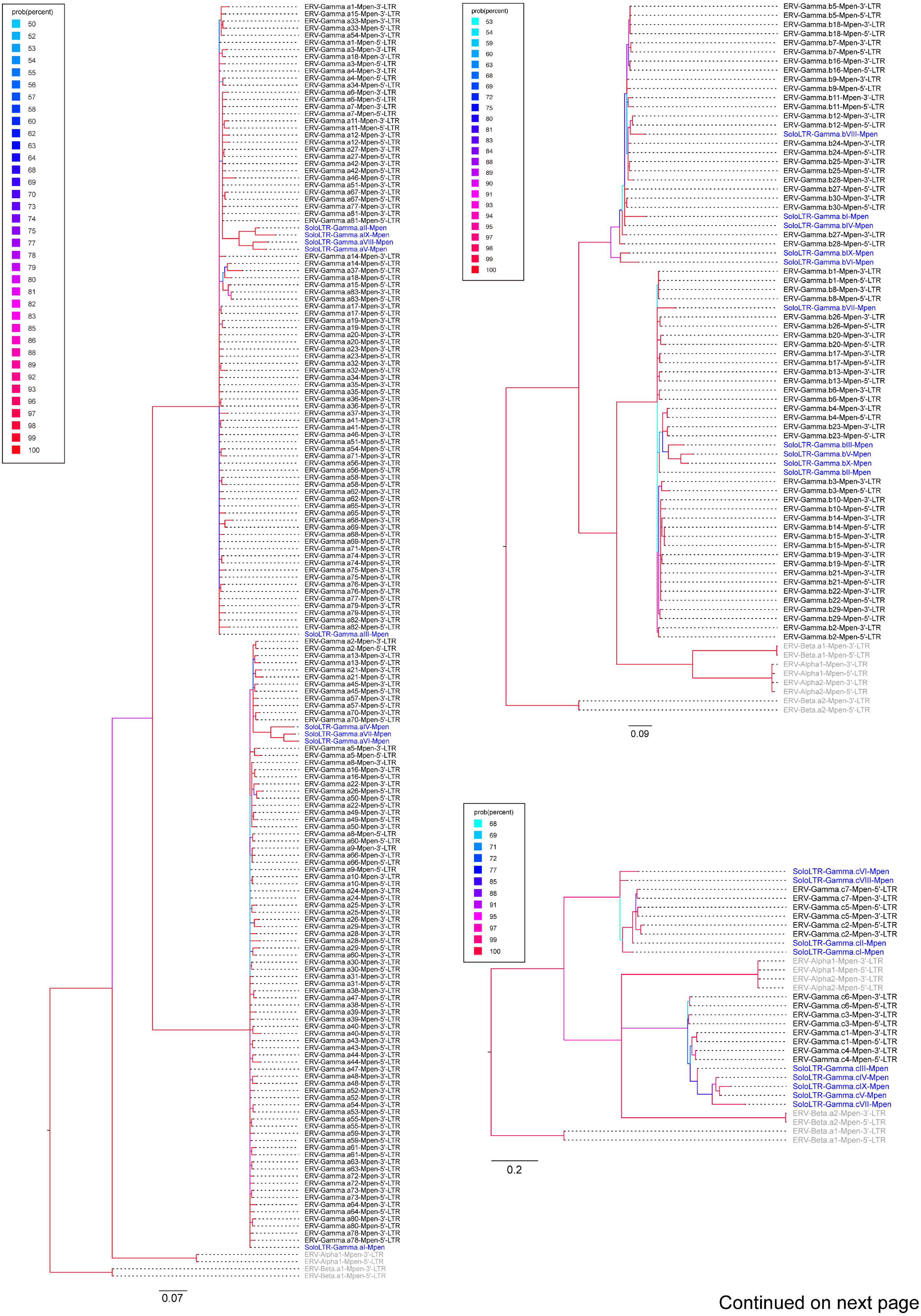

Continued on next page

Continued from previous page

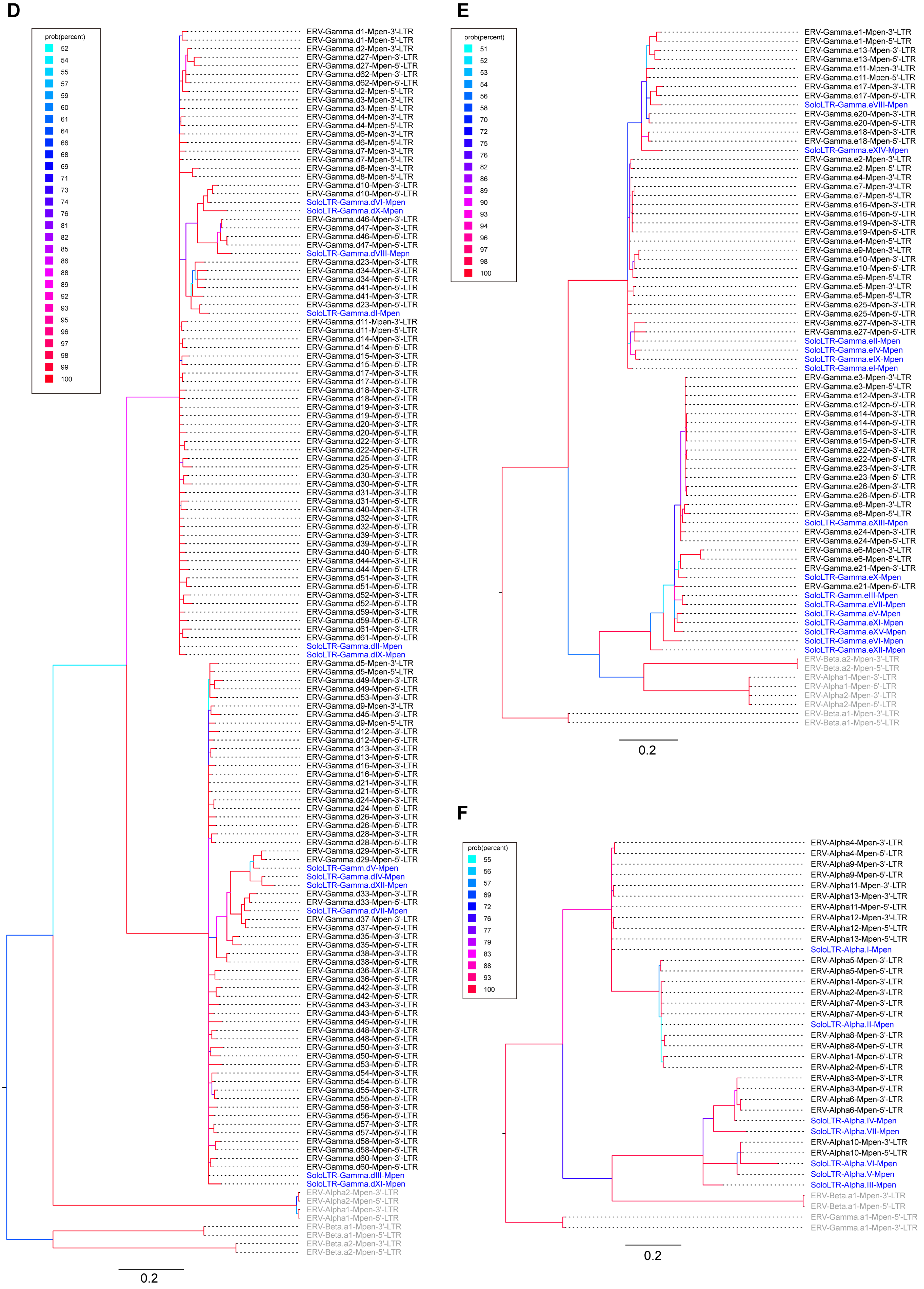

Continued on next page

Continued from previous page

G

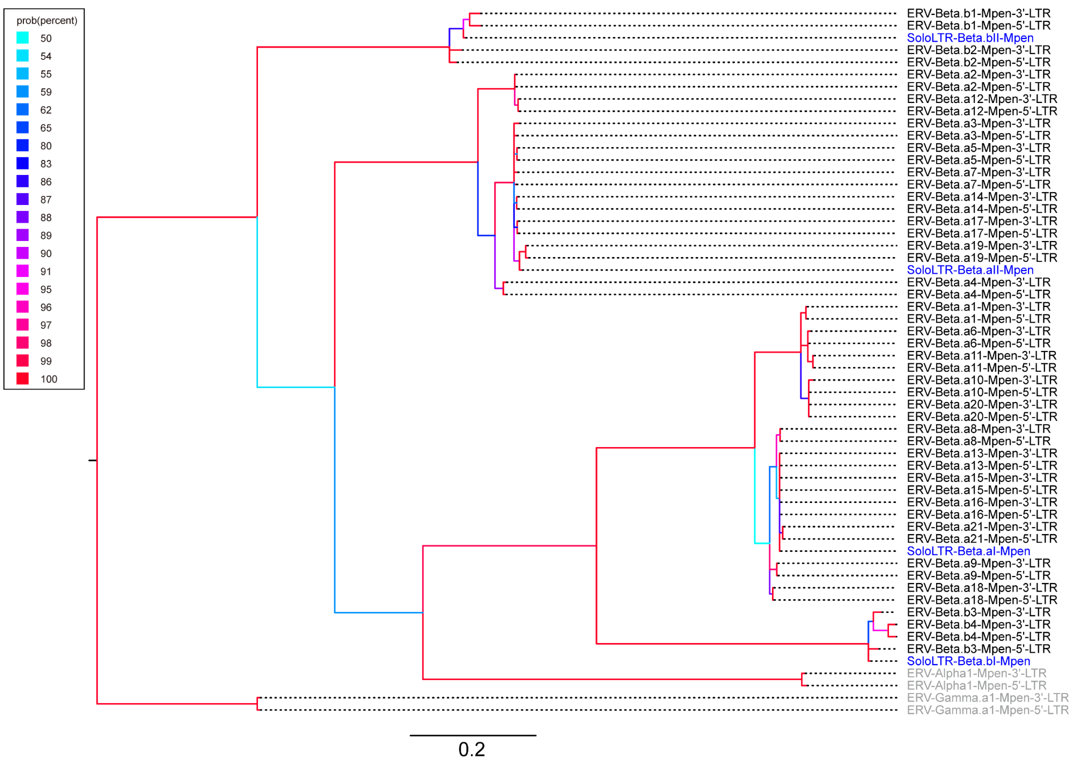

Fig. S5. Nucleotide phylogenetic trees of paired long terminal repeats (LTRs) and Solo LTRs from various MPERV families in the Chinese pangolin. Trees are shown for the: (A) ERV-Gamma.a-Mpen family; (B) ERV-Gamma.b-Mpen family; (C) ERV-Gamma.c-Mpen family; (D) ERV-Gamma.d-Mpen family; (E) ERV-Gamma.e-Mpen family; (F) ERV-Alpha-Mpen family; (G) ERV-Beta.a-Mpen and ERV-Beta.b-Mpen families. Phylogenetic trees were constructed using Bayesian inference based on nucleotide sequences of paired LTRs from individual MPERV insertion sites and consensus sequences of Solo LTRs for each lineage. The scale bar represents nucleotide substitutions per site. Posterior probability values at nodes are indicated using a color gradient according to the legend. All Solo LTR sequences are labeled in blue text. Each tree was rooted using paired LTRs from other ERV families, which are labeled in gray text.

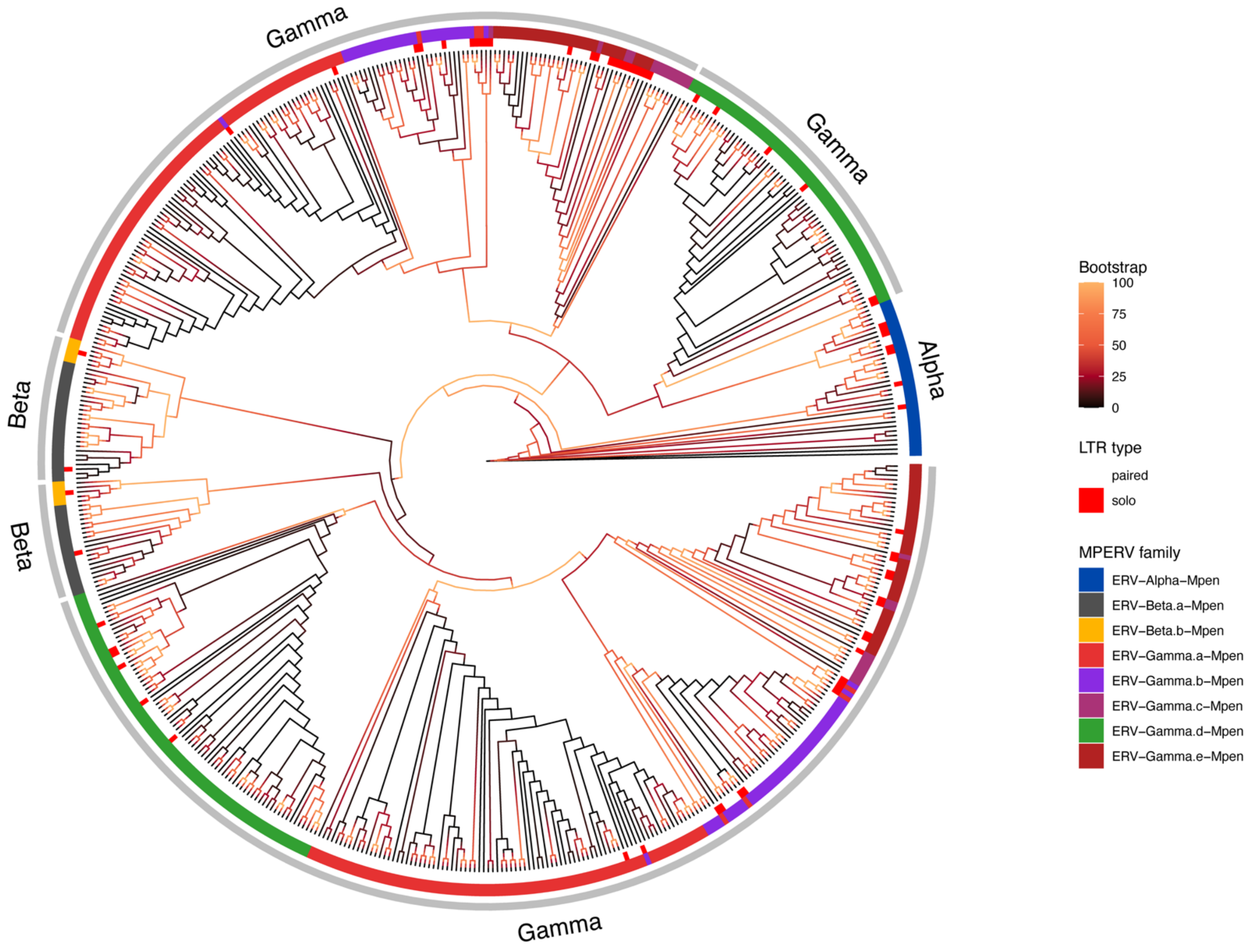

Fig. S6. Phylogenetic tree of MPERV LTRs. This tree was constructed using the maximum likelihood method based on nucleotide sequences of paired LTRs from individual MPERV insertion sites and consensus sequences of Solo LTRs for each lineage. To enhance topological clarity, branch lengths are not drawn to scale and the tree emphasizes only the branching pattern. Bootstrap support values for nodes are represented by a color gradient. The innermost ring indicates the positions of Solo LTRs using red shading. The middle ring annotates the clustering of LTRs (including Solo LTRs) from distinct lineages within the phylogeny. The outermost ring annotates the clustering of LTRs categorized into three families, along with Solo LTRs.
